# Mitochondrial energy metabolism correlates with an immunosuppressive tumor microenvironment and poor prognosis in esophageal squamous cell carcinoma

**DOI:** 10.1016/j.csbj.2023.08.022

**Published:** 2023-08-24

**Authors:** Zewei Zhang, Gaowa Jin, Juan Zhao, Shuqin Deng, Feng Chen, Gaowa Wuyun, Lei Zhao, Quanfu Li

**Affiliations:** a Department of Radiation Oncology, Sun Yat-sen University Cancer Center, Guangzhou, China; b State Key Laboratory of Oncology in South China, Guangzhou, China; c Collaborative Innovation Center for Cancer Medicine, Guangzhou, China; d Department of Medical Oncology, Ordos Central Hospital, Ordos, China

**Keywords:** Mitochondrial metabolism, Single-cell, Tumor microenvironment, Crosstalk, Esophageal squamous cell carcinoma

## Abstract

**Background:**

Reprogramming of mitochondrial energy metabolism (MEM) is an important hallmark of tumorigenesis and cancer progression. Currently, there are no studies that have examined MEM in the tumor microenvironment (TME) of esophageal squamous cell carcinoma (ESCC), and relevant drug targets have not yet been identified.

**Methods:**

The ESCC single-cell transcriptome sequencing dataset, GSE145370, was analyzed, using the AUCell R package to screen for MEM-related genes in high-scoring cell populations. Monocle was used to infer cell differentiation and CellChat to analyze intercellular communication networks. Finally, transcription levels of prognostic genes were analyzed using a complementary DNA microarray from 15 patients with ESCC.

**Results:**

A total of 121 MEM-related genes were differentially expressed in seven cell populations in the TME, and four high-scoring cell populations were identified. As a result, the MEM state of T cells is significantly different from that of macrophages and epithelial cells, and signaling communication between T cells and macrophages is the strongest. These findings suggest that immunosuppression is related to metabolic reprogramming. Additionally, marker genes of high-scoring cells and the top10 receptor-ligand pairs may become new targets for rebuilding immune cell metabolism. Furthermore, the 4-MEM gene risk signature had good predictive power for overall survival and drug sensitivity. MAP1LC3A, APOE, APPL1, and NDUFA are novel potential immunotherapeutic targets for remodeling the TME. Finally, teal-time quantitative PCR was used to verify APOE and MAP1LC3A expression.

**Conclusion:**

MEM heterogeneity was observed in the immunosupressive TME of ESCC. Prognostic models based on MEM-related genes are helpful for screening early treatment patient groups and realizing personalized treatment. APOE and MAP1LC3A are potential target genes for the development of anti-ESCC drugs based on MEM-related genes.

## Highlights

• 121 marker mitochondrial energy metabolism-related genes in ESCC microenvironment.
• 4 high-scoring cell populations were discovered in ESCC microenvironment.
• Mitochondrial energy metabolism of T cells is different from that of macrophages.
• Top10 receptor-ligand pairs may be new targets for rebuilding immune cell metabolism
• A 4-MEMRGs prognostic signature could predict drug sensitivity.

## Graphical Abstract

**Figure:**
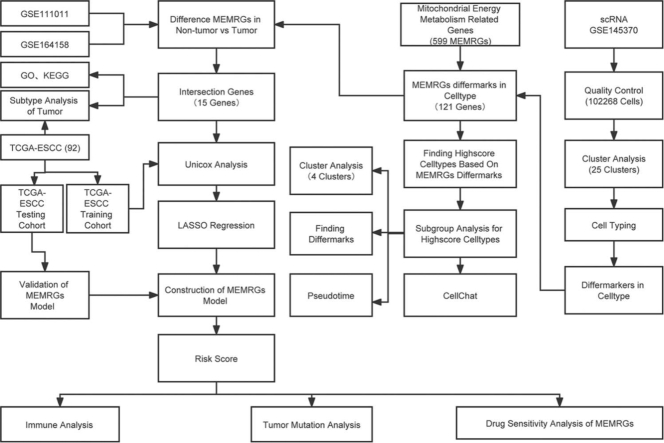

## 1 Introduction

Esophageal cancer (ESCA) ranks ninth in incidence among common cancers and sixth in number of cancer-related deaths [1]. In China, esophageal squamous cell carcinoma (ESCC) accounts for approximately 86% of all ESCA cases. Unfortunately, only 20–30% patients with ESCC benefit from treatment with immune checkpoint inhibitors [2]. This may be due to insufficient metabolic reprogramming of the immunosuppressive tumor microenvironment (TME), limiting the viability of antitumor immunity [3]. Specifically, metabolic reprogramming promotes defective mitochondrial oxidative phosphorylation. Dysfunctional mitochondria accumulate in tumor-infiltrating lymphocytes, leading to hypometabolism, exhaustion, and dysfunctional T cells [4], [5], [6]. Therefore, understanding the changes in mitochondrial energy metabolism (MEM) in the TME and their influence on immune cells will help identify new ways to rebuild immune cell metabolism and improve immunotherapy.

To sustain rapid cell proliferation, tumor cells activate and/or modify metabolic pathways to gain more energy through a process known as MEM reprogramming. This dynamic process occurs during tumorigenesis and development. Indeed, the flexibility of MEM can meet the needs of various stages of tumorigenesis and metastasis [7]. For example, MEM adaptation promotes epithelial-mesenchymal transition, a multifactorial cellular state involving the loss of adhesion and enhanced migratory capacity. This increases migration and metastasis [8]. Mitochondrial metabolism-dependent macromolecular synthesis and oncogenic metabolite production support tumor growth. The resulting metabolic intermediates can be funneled into various biosynthetic pathways required for cell proliferation, such as the pentose phosphate pathway involved in NADPH synthesis and the one-carbon metabolic pathway involved in nucleotide synthesis [9], [10]. Moreover, the mitochondrial electron transport chain can meet the bioenergetic and biosynthetic demands of tumor cells because it can oxidize ubiquinol to ubiquinone. This is necessary for maintaining Krebs cycle function and essential enzyme activity for pyrimidine synthesis [10]. Gong et al. found that inducing mitochondrial oxidative phosphorylation dysfunction could increase the accumulation of reactive oxygen species (ROS), inhibiting proliferation and inducing apoptosis in ESCC cells [11]. Therefore, mitochondrial energy metabolism pathways not only affect the occurrence and development of ESCC, but are also potential targets for ESCC therapy.

This study explored the expression of MEM-related genes in different cell populations of the ESCC microenvironment using single-cell RNA sequencing data. It also revealed the molecular interactions that may affect the metabolic state of immune cells. Next, we constructed an MEM prognostic model and validated the expression of four identified prognostic genes using qPCR. This study is the first in the field of ESCC to explore the MEM status of different cells in the TME and investigate the prognostic significance of MEM-related genes. These findings will help identify new targets for remodeling immune cell metabolism and benefit clinicians for accurate prognostic assessment and personalized treatment of patients with ESCC.

## 2 Methods and materials

### 2.1 Data download

The expression profile datasets GSE111011 and GSE164158 [12] were downloaded from the Gene Expression Omnibus (GEO) database (https://www.ncbi.nlm.nih.gov/geo/) [13]. GSE111011 included seven ESCC cases and their paired paracancerous tissue samples. GSE164158 included eight ESCC cases and paired paracancerous tissue samples. Additionally, we also downloaded the ESCC single-cell transcriptome sequencing dataset GSE145370 [14], which included seven ESCC cases and their adjacent tissue samples (Supplementary Table S1). The normal samples in GSE164158 comprised six males and two females, whereas the ECSS samples included six males and two females. Unfortunately, the GSE111011 and GSE145370 datasets did not provide detailed information regarding the age and sex of the patients.

The TCGAbiolinks R package (version 2.22.4) [15] was used to download the FPKM (Fragments Per Kilobase of transcript per Million mapped reads) expression profile data, count data matrix, survival data, and clinical data of 185 patients with ESCA from The Cancer Genome Atlas (TCGA) Genomic Data Commons (GDC) website (https://portal.gdc.cancer.gov/). Complete clinical and survival data were available for 161 patients, of which 92 patients with ESCC were included in follow-up analyses (Supplementary Table S2). At the same time, the patients’ somatic cell copy number variation (CNV) data were downloaded, and "Masked Somatic Mutation" was selected for the somatic mutation data. Then, we used the maftools R-package (version 2.14.0) [16] to visualize somatic mutations and obtain tumor mutation burden (TMB) [17], microsatellite instability score (MSI) [18], and Tumor Immune Dysfunction and Exclusion (TIDE) [19] data.

In addition, human-related MEM-related pathways were searched for in the Kyoto Encyclopedia of Genes and Genomes (KEGG) database [20], [21], [22] using the keyword "mitochondrial energy metabolism,” obtaining the hsa04152, hsa04371, hsa05010, and hsa04211 signaling pathways. Finally, the rjson R package (version 0.2.21) was used to extract 599 MEM-related genes from these four signaling pathways (Supplementary Table S3).

### 2.2 Quality control of single-cell data using Seurat

The Seurat (version 4.3.0) R package [23] was used to import the count matrix and clinical single-cell sequencing data of the seven patients with ESCC from the GSE145370 dataset. Cell homeostasis can be determined by the proportion of mitochondrial genes in the total genetic material. When a cell has a higher ratio of mitochondrial genes to all genes, it may be in a state of stress [24], [25]. Therefore, we filtered cells with > 10% mitochondrial gene content. In addition, we filtered low-quality cells according to the quality control criteria “0 < nFeature_RNA < 3000″, which was different from the criteria "nFeature_RNA > 400" used in the original study[14]. The criteria was not strictly regulated, highly subjective, and mainly related to the number of genes detected in all cells.

Data were normalized using log normalization. The identified genes with high variability in individual cells were adjusted for the relationship between mean expression and dispersion. Principal components were identified using Principal Component Analysis (PCA) with variable genes as inputs. The top 20 principal components were selected as inputs for t-distributed stochastic neighborhood embedding (t-SNE). To visualize the cell clustering results, we used the t-SNE method, which is different from the UMAP method used in the original study of GSE145370 [14]. t-SNE tends to produce more visually separated clusters than UMAP, making it easier to interpret and identify distinct cell populations. t-SNE is well-suited for capturing nonlinear relationships in data. This is critical for visualizing complex cellular heterogeneity.

### 2.3 Single-cell data clustering and annotation

Cell clustering was first performed using the FindClusters function with a 0.5 resolution parameter. A total of 25 clusters were identified. Then, standard cell-type annotations were performed using the HumanPrimaryCellAtlasData dataset from the SingleR (version 2.0.0) R package [26], and FindAllMarkers was used to find differentially expressed genes (DEGs) between cell populations. The DEGs were intersected with the identified MEM-related genes and sorted according to the average fold change (avg_log2FC) among the cells, and the resulting top20 MEM-related genes were selected for correlation analysis. When performing cell annotation, the original study used cell marker genes reported previously in the literature for annotation [14], while this study used SingleR for annotation. The original study focused on immune cells in ESCC single-cell data and the expression of previously reported marker genes in different cell types, and did not screen DEGs in different cell types using avg_log2FC. However, this current study focused on MEM-related genes; therefore, their expression was observed, revealing 121 differentially expressed MEM-related genes between the different cell types (P < 0.05).

### 2.4 Pseudochronological analysis of cell subpopulations

Cell differentiation was determined using the Monocle (2.26.0) R package [27]. A cell dataset was constructed using integrated gene expression matrices exported from the Seurat objects to Monocle. Variable genes were defined using the “dispersionTable” function, and the cell order was determined using the “setOrderingFilter" function. Finally, the ”reduceDimension” function was used to reduce the dimensionality. The cells were arranged along the trajectory using the “OrderCell” function. The differentiation times of the cell subpopulations were mapped based on clustering properties and marker gene analysis.

### 2.5 Analysis of intercellular communication

Pairs of intercellular receptors and ligands between the two cell types were analyzed using the CellChat R package (1.6.1). The cell type and gene expression information from the Seurat object were used to create a Cellchat object. The CellChatDB human database was employed for preprocessing and cell communication network inference.

### 2.6 Difference analysis based on MEM marker genes in GEO data

To determine whether MEM-related gene expression varied based on transcription, the Wilcoxon test was used to compare expression of 121 MEM-related genes in cancerous tissue samples with those in paracancerous tissue samples from GSE111011 and GSE164158. The screening conditions for the DEGs were |log2FC= > 0 and FDR (adjusted P-value) < 0.05, and a volcano map was drawn. The intersection of the differential MEM-related genes were obtained from the two datasets, GSE111011 and GSE164158, and used to identify the key MEM-related genes and draw a heat map to display their gene expression.

### 2.7 Molecular typing of key genes in tumor samples based on MEM

The ConsensusClusterPlus (version 1.58.0) R package [28] was used to perform consensus clustering on 92 ESCC tumor samples from TCGA-ESCA based on key MEM-related genes. Spearman’s correlation was used to calculate the distance, and the clustering algorithm used was partitioning around medoids (PAM).

### 2.8 Prognostic model construction and validation

To further determine the influence of key MEM-related genes on ESCC prognosis, they were used as candidate genes to construct a prognostic model. In this study, the caret (version 6.0–93) R package was used to divide the TCGA-ESCC dataset into training and validation sets at a 3:2 ratio. The model was constructed based on 56 samples from the training set and verified using 36 samples from the validation set. First, a univariate COX regression analysis was conducted. Next, we analyzed the genes that had a significant impact on prognosis (p value < 0.3). The obtained genes were subjected to LASSO (Least Absolute Shrinkage and Selection Operator) regression analysis. A risk model was constructed using multivariate Cox regression analysis to obtain a Risk Score for each tumor sample.

$$\mathrm{riskScore}=\sum_{i}\mathrm{Coefficient}\left({\mathrm{gene}}_{i}\right)*\mathrm{mRNAExpression}({\mathrm{gene}}_{i})$$

Based on the median risk score, patients were categorized into high- and low-risk groups. Kaplan-Meier and time-dependent receiver operating characteristic (ROC) curve analyses were used to evaluate the accuracy of the risk model. This model was used to calculate the risk score for each patient in the validation set.

### 2.9 Complementary DNA (cDNA) microarray of ESCC tissues

A cDNA microarray of ESCC tissues (MecDNA-HEsoS030PG01) was purchased from the Shanghai Outdo Biotech Company (Shanghai, China). It contained 15 ESCC tissues and corresponding adjacent noncancerous tissues, along with patient clinical data, including sex, age, lesion size, histological grade, and lymphatic metastasis status. Detailed clinicopathological data of the specimens in this cDNA microarray are listed in Supplementary Table S4. The use of the cDNA microarray was approved by the above company's ethics committee (approval number: YB M-05–02).

### 2.10 Quantitative real-time PCR (RT-qPCR)

Total RNA was extracted from ESCC and adjacent noncancerous tissues using the TRIzol reagent (Sigma, St. Louis, MO, USA). Subsequently, RNA was reverse transcribed to cDNA using the PrimeScript™ RT Master Mix (Perfect Real Time) (TaKaRa, Dalian, China) according to the manufacturer's instructions. RT-qPCR was performed using the ChamQ Universal SYBR qPCR Master Mix kit (Vazyme, Nanjing, China) on a Roche LightCycler® 480II PCR system. Primers used are listed in Supplementary Table S5.

### 2.11 Mutation analysis

The somatic data of 92 patients with TCGA-ESCC was downloaded and the maftools (version 2.14.0) R package [16] was used for statistics analysis. First, the “subsetMaf” function was used to construct two mutation annotation format (MAF) files for the high- and low-risk groups, the “mafCompare" function was used to compare differentially mutated genes, and the oncoplot function was used to draw a waterfall diagram. Then, mutation signature analysis was performed on tumor samples using the deconstructSigs (version 1.8.0) R package and the top eight mutation signatures were visualized.

### 2.12 Copy number alteration (CNV) analysis

GISTIC 2.0 analysis was performed on the downloaded CNV fragments data using GenePattern [29]. All parameters were analyzed using the default settings provided by GISTIC 2.0. Finally, the maftools package (version 2.14.0) [16] was used to visualize these results.

### 2.13 Single sample gene set enrichment analysis (ssGSEA)

Next, 16 immune cell types (Supplementary Table S6) and 13 gene sets related to immune pathways (Supplementary Table S7) were extracted from the literature [30], and the ssGSEA method in the GSVA (version 1.46.0) package was used to analyze the degree of tissue enrichment [31].

### 2.14 Statistical analysis

R (version 4.2.2) was used to process and analyze the data. For the comparison of two groups of continuous variables, the independent Student's t-test was used for normally distributed variables, and the Wilcoxon rank sum test was used for non-normally distributed variables. Two groups of categorical variables were compared and statistically analyzed using the chi-square or Fisher's exact tests. The survival package in R (version 3.4–0) was used for survival analysis. The log-rank test was used to examine the significance of the survival differences between the two groups. Receiver operating characteristic (ROC) curves were plotted using the timeROC package (version 0.4). Independent prognostic factors were identified using univariate and multivariate Cox analyses. All statistical P-values were two-sided, and P < 0.05 was considered statistically significant.

## 3 Results

### 3.1 Overall study design flowchart

Fig. S1 outlines the analysis of the ESCC single-cell transcriptome dataset (GSE145370), including the identification of MEM-related genes, inference of cell differentiation, analysis of intercellular communication network, and assessment of prognostic gene using cDNA microarrays from patients with ESCC.

### 3.2 Single-cell data reveal the cellular heterogeneity of ESCC

**Fig. 1:**
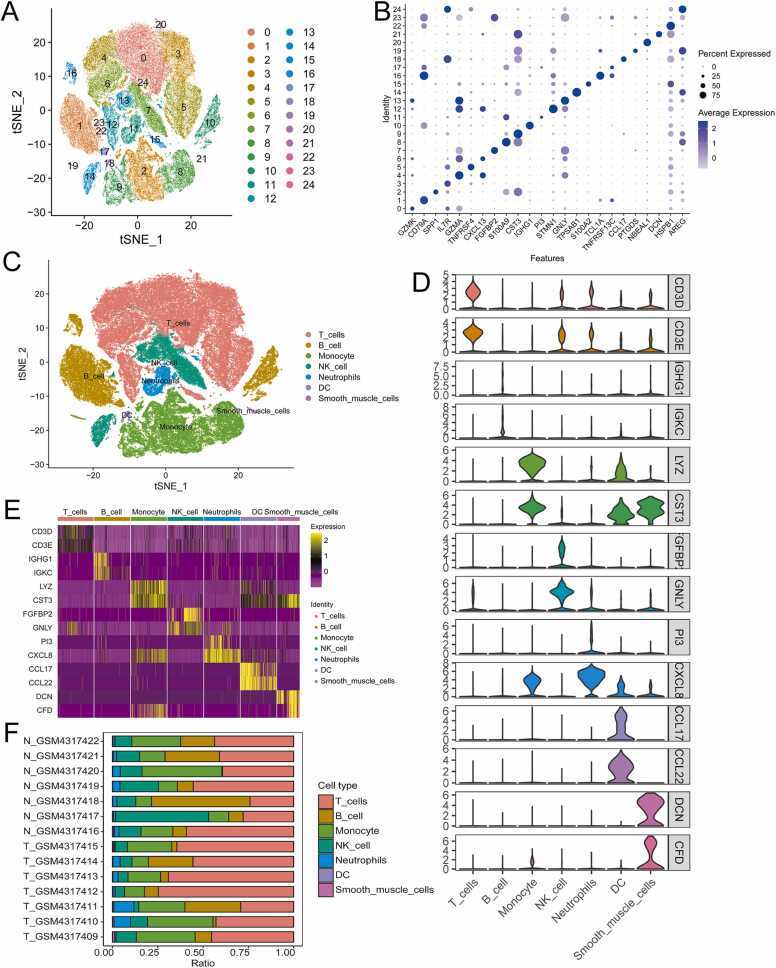
Dimensionality reduction cluster analysis and feature description of single-cell sequencing data. **(A)** Dimensionality reduction cluster analysis. **(B)** The expression of the top1 marker gene in each cell cluster. **(C)** t-SNE distribution in different cell types. **(D)** Violin plot showing the top2 differential genes in each cell type. **(E)** Heatmap showing the top2 differential genes in each cell type. **(F)** Proportion of each cell population in different samples. DC, dendritic cells; t-SNE, t-distributed stochastic neighborhood embedding.

In this study, single-cell RNA sequencing (scRNA-seq) data from 14 cases using GSE145370 (seven cases of cancer tissues and seven cases of paracancerous tissues) was analyzed. A total of 102,268 filtered cells were obtained following the quality control procedures. The SNN (Spiking Neural Network) algorithm was used for cluster analysis, obtaining 25 optimal cell clusters (Fig. 1A). The top1 marker genes in the 25 optimal cell populations were identified using bubble plots (Fig. 1B). These marker genes were highly expressed in the corresponding cell clusters, indicating cluster-specific expression patterns and cell types.

The cell types in each cluster were identified using the SingleR package (Fig. 1C). A total of 50,113 cells in clusters 0, 3, 4, 5, 6, 12, 15, and 20 were annotated as T cells, accounting for 49.002% of the total number of cells. A total of 16,280 cells in clusters 1, 10, 16, 17, and 22 were annotated as B cells, accounting for 15.919% of the total number of cells. Clusters 2, 8, 9, and 19 were annotated as monocytes (21075; 20.608%). A total of 10,773 cells in clusters 7, 13, 14, 23, and 24 were annotated as natural killer (NK) cells, accounting for 10.534% of the total number of cells. Additionally, cluster 11 was annotated as neutrophils (3426; 3.350%), cluster 18 was annotated as dendritic cells (DCs) (413; 0.404%), and cluster 21 was annotated as smooth muscle cells (188; 0.184%). These results demonstrate the diversity of cell types in the dataset and reveal the existence of cellular heterogeneity. Violin plots were used to show the top2 differential genes in the various cell types (Fig. 1D). The results showed that, compared to their expressions in other cells, *CD3D* and *CD3E* were highly expressed in T cells, *IGHG1* and *IGKC* were highly expressed in B cells, *LYZ* and *CST3* were highly expressed in monocytes, *FGFBP2* and *GNLY* were highly expressed in NK cells, *PI3* and *CXCL8* were highly expressed in neutrophils, *CCL17* and *CCL22* were highly expressed in DCs, and *DCN* and *CFD* were highly expressed in smooth muscle cells. Furthermore, the expression of each cell's top2 differential genes were displayed on a heat map. When predicted by violin plots, the results were similar (Fig. 1E, Supplementary Table S8). A stacked histogram was used to visualize the proportion of cell types in each sample (Fig. 1F). NK cells were present in lower numbers in tumor tissues than in non-tumor tissues.

### 3.3 Expression of MEM-related genes in different cell populations of the ESCC TME

**Fig. 2:**
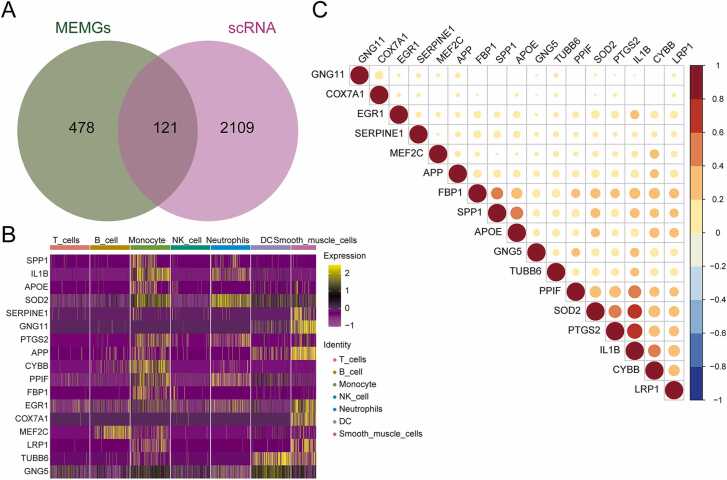
Correlation analysis of marker MEM genes based on GSE145370. **(A)** The Venn diagram shows the number of intersections between mitochondrial energy-related genes and differential genes between cell groups, and the area where the two circles overlap is marker MEM genes. **(B)** Heatmap showing the expression of top20 marker MEM genes in each cell type. **(C)** Correlation heatmap showing the expression correlation of top20 marker MEM genes in cells. MEM, mitochondrial energy metabolism.

In this study, the 599 MEM-related genes identified were intersected with the DEGs of different cell populations to obtain 121 marker MEM-related genes (Fig. 2A). These 121 MEM-related genes were sorted according to the average log2 fold change (avg_log2FC) to potentially identify genes that exhibited significant differential expression in different cell states or populations. The top20 MEM-related genes included the following 17 genes: *SPP1, IL1B, APOE, SOD2, SERPINE1, GNG11, PTGS2, APP, CYBB, PPIF, FBP1, EGR1, COX7A1, MEF2C, LRP1, TUBB6,* and *GNG5*. According to the heat map, the expression levels of these 17 genes differed significantly among the various cell types (P < 0.05, Fig. 2B). *SPP1, IL1B, APOE, PTGS2, CYBB, PPIF,* and *FBP1* were highly expressed in monocytes and lowly expressed in T, B, and NK cells. *SOD2* and *GNG5* were highly expressed in monocytes, neutrophils, and DCs. In addition, the correlation among the 17 MEM-related genes was shown using a correlation heat map (Fig. 2C, Supplementary Table S9). Significant correlations were found between *PTGS2* and *IL1B* (r = 0.680, P < 0.05), *IL1B* and *SOD2* (r = 0.605, P < 0.05), *IL1B* and *PPIF* (r = 0.483, P < 0.05), *PTGS2* and *SOD2* (r = 0.480, P < 0.05), and *SPP1* and *APOE* (r = 0.473, P < 0.05).

### 3.4 Analysis of cell subgroups with enriched expression of marker MEM-related genes

**Fig. 3:**
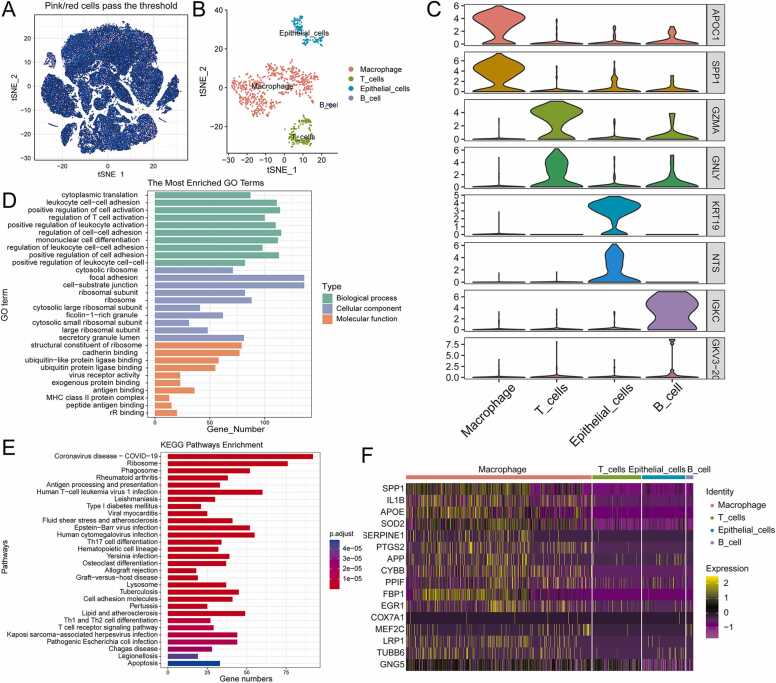
Analysis of high-scoring cell populations based on marker MEM genes. **(A)** t-SNE shows the distribution of high-scoring cell populations in all cells. **(B)** t-SNE distribution of different cell types in the high-scoring cell population. **(C)** The top2 differential genes of each cell type in the high-scoring cell population are shown by violin plot. **(D-E)** Histogram showing GO and KEGG analysis results of differential genes in high-scoring cell populations, respectively. **(F)** Heatmap showing the expression of marker MEM genes in different cell types of high-scoring cell populations. GO, Gene Ontology; KEGG, Kyoto Encyclopedia of Genes and Genomes; MEM, mitochondrial energy metabolism; t-SNE, t-distributed stochastic neighborhood embedding.

In this study, AUCell was used to explore the enrichment fraction of MEM-related marker genes, and cells with an enrichment score higher than 0.086 were defined as "high-scoring cells". As a result, 940 high-scoring cells were obtained and the distribution of high-scoring cell populations among all cells was visualized using t-SNE (Fig. 3A). Then, the SNN algorithm was used for cluster analysis and the clustering of high-scoring cell subgroups was performed using t-SNE based on the dimensionality reduction results of the PCA (Fig. 3B). SingleR package was used to identify different cell types and find their DEGs (Supplementary Table S10). The high-scoring cell populations included macrophages (613, 65.213%), T cells (161, 17.128%), epithelial cells (143, 15.213%), and B cells (23, 2.447%) (Fig. 3B). The top2 differential marker genes in the various cell types were identified by drawing a violin plot (Fig. 3C). The DEGs between different cells were enriched (P < 0.05, q<0.05) using Gene Ontology (GO) (Fig. 3D, Supplementary Table S11) and KEGG (Fig. 3E) analyses with the clusterProfiler R package. The results of the GO analysis revealed that the primary enriched biological processes of high-scoring cell populations were those related to cell activation, such as positive regulation of cell activation, regulation of T cell activation, and positive regulation of leukocyte activation. The primary enriched cell components were related to ribosomes, such as cytosolic ribosome, ribosomal subunit, and ribosome. Finally, the primary enriched molecular functions were related to immunity, such as exogenous protein binding, major histocompatibility complex (MHC) class II protein complex, and peptide antigen binding (Fig. 3D, Supplementary Table S11). Furthermore, KEGG pathway analysis also showed that the DEGs of high-scoring cell populations were primarily enriched in inflammation, immunity, and other related signaling pathways, such as Th17 cell differentiation, Th1 and Th2 cell differentiation, and the T cell receptor signaling pathway (Fig. 3E). In addition, a heat map was used to show the expression of 17 MEM-related marker genes of the avg_log2FC top20 among high-scoring cell populations (Fig. 3F). As shown in Fig. 3F, the expression of the 17 MEM-related genes in macrophages was significantly higher than that in other cell types (P < 0.05).

### 3.5 Pseudo-chronological analysis of high-scoring cell populations

**Fig. 4:**
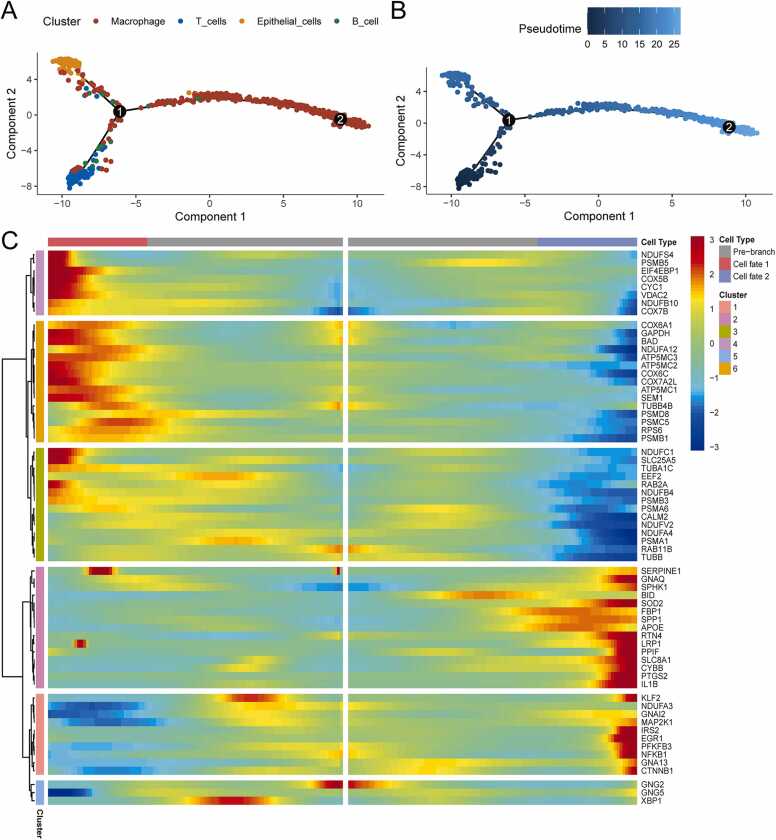
Pseudo-chronological analysis of high-scoring cell populations. **(A)** Pseudochronogram based on high-scoring cell populations. **(B)** Pseudo-time trajectory map. **(C)** With 1 as the node, the dynamic heat map of differential MEM genes between Cell state1 and Cell state2 in the pseudo-chronological branch. MEM, mitochondrial energy metabolism.

Using Monocle R, pseudo-chronological analyses were performed on high-scoring cell populations, and a trajectory map was constructed for pseudo-time. The pseudo-chronological diagram is colored from two aspects of the cell population process (Fig. 4A) and pseudo-time course (Fig. 4B), and different branches pass along the direction of the pseudo-chronological sequence. The differences in the marker MEM-related genes between the different clades were examined using the BEAM function, highlighting 65 genes that were different between the two clades. These results were visualized on a dynamic heat map (Fig. 4C). From the first node to the root is pre-branch, which is the state corresponding to macrophages; Cell fate1 is the state corresponding to T cells and Cell fate2 contains the state corresponding to epithelial cells and macrophages. Considering their expression levels, the differential marker MEM-related genes were classified into six distinct categories based on the similarities in their expression patterns across different cell states. This not only showed the process of the cell population, but also the process of pseudotime. Through the direction of the pseudochronology, it was observed that the cell population experienced different branches. These results provide information on cell state changes and MEM dynamics during quasi-chronological processes.

### 3.6 Cell communication among high-scoring cell populations

**Fig. 5:**
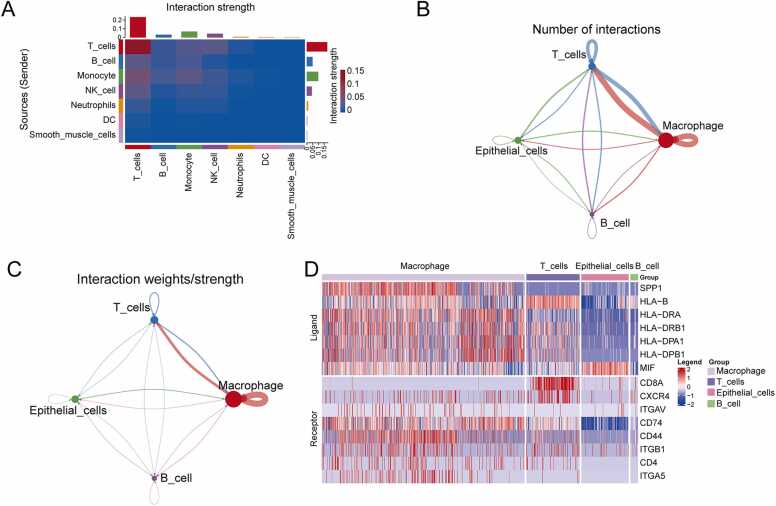
Cell-to-cell communication analysis of high-scoring cell populations. **(A)** Heatmap of the interaction strength between all cell types. **(B)** Network diagram of the number of interactions between different cell types in high-scoring cell populations. **(C)** Network diagram of interaction weights between different cell types in high-scoring cell populations. **(D)** Expression of some ligands and receptors in high-scoring cell populations.

First, the strength of interactions among all cell populations were visualized using a heatmap (Fig. 5A). Each cell type on the ordinate is a signal sender. The strength of their communication with other cell types on the abscissa is shown in the top and right columns. Each histogram represents the sum of the communication intensities of the corresponding cell types. T cells communicated most strongly with other cell types, either as ligand cells (weight sum = 0.170) or recipient cells (weight sum = 0.230).

The interactions between different cell types in the high-scoring cell population were also analyzed. The communication network diagram shows the number of interactions between different cell types (Fig. 5B) and the strength of the interaction signals (Fig. 5C). There were 68 interactions between macrophages, 71 interaction pairs between macrophages as ligand cells and T-cells, and 49 interaction pairs between macrophages as recipient cells and T-cells. The results of the interaction signal strength analysis demonstrated that the strength of the interactions between macrophages themselves were the strongest (weight = 0.133), the interaction strength between macrophages as ligand cells and T cells was the strongest (weight = 0.053), and the strength between macrophages as recipient cells and T cells was the strongest (weight=0.022). In addition, there were 319 pairs of ligand-receptors in the high-scoring cell populations (Supplementary Table S12). This study employed a heat map to display the 10 receptor and ligand pairs with the highest communication probability (Fig. 5D), including "SPP1 - CD44,” "MIF - (CD74 +CD44),” "HLA-DRA - CD4,” "MIF - (CD74 +CXCR4),” "HLA-DPA1 - CD4,” "HLA-DPB1 - CD4,” "HLA-B - CD8A,” "SPP1 - (ITGA5 +ITGB1),” "SPP1 - (ITGAV +ITGB1),” and "HLA-DRB1-CD4.”

### 3.7 Differential analysis based on marker MEM-related genes in transcriptome data

**Fig. 6:**
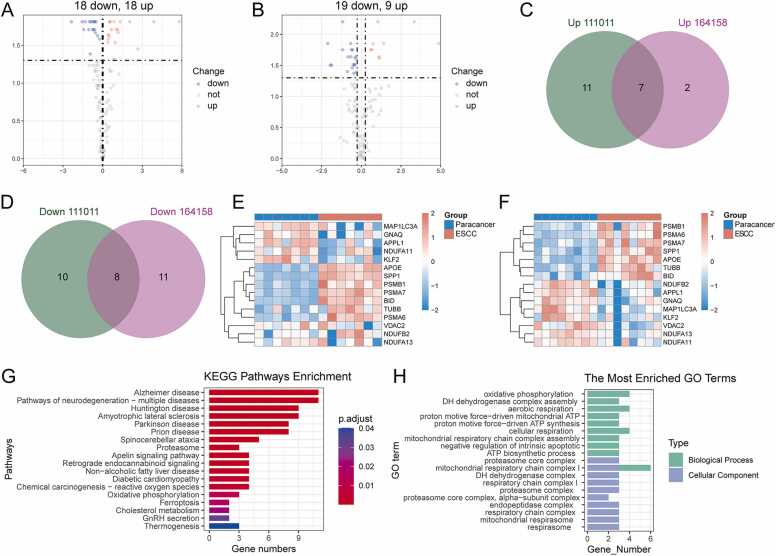
Transcriptome analysis of MEM genes in GEO datasets. **(A-B)** Volcanic maps showing the differential expression of MEM genes in cancer tissues in the GSE111011 and GSE164158 datasets, respectively. **(C-D)** Venn diagrams showing intersecting genes that are up-regulated and down-regulated in both datasets, respectively. **(E-F)** Heatmaps showing the expression of 15 differential MEM genes in cancer and adjacent tissues in the GSE111011 and GSE164158 datasets, respectively. **(G)** GO enrichment analysis of key MEM genes. (H) KEGG enrichment analysis of key MEM genes. GEO, Gene Expression Omnibus; MEM, mitochondrial energy metabolism.

First, the expressions of 121 MEM-related marker genes in ESCC and paracarcinoma were explored using the GSE111011 dataset, 36 of which differed between ESCC and adjacent tissues (Supplementary Table S13). As displayed in the volcano plot, 18 genes were upregulated and 18 genes were downregulated among the 36 DEGs (Fig. 6A). Subsequently, the expressions of the 121 MEM-related genes in ESCC and para-cancerous tissues were explored using the GSE164158 dataset, of which 28 differed between ESCC and paracancerous tissues (Supplementary Table S14). The volcano plot showed that among the 28 DEGs, 9 were upregulated and 19 were downregulated (Fig. 6B). Seven genes were upregulated in both datasets (Fig. 6C), including *TUBB, PSMB1, PSMA7, PSMA6, BID, APOE,* and *SPP1*, while eight genes were downregulated in both datasets (Fig. 6D), including *NDUFB2, VDAC2, NDUFA13, NDUFA11, GNAQ, MAP1LC3A, APPL1,* and *KLF2*. Overall, heat maps showed that 15 genes were significantly different between ESCC and para-cancer tissues in the GSE111011 (P < 0.05, Fig. 6E) and GSE164158 (P < 0.05, Fig. 6F) datasets.

To explore the molecular mechanisms involved, GO enrichment analysis of the 15 key MEM-related genes was performed using the clusterProfiler R package (Supplementary Table S15). In the GO enrichment results, each term is arranged in ascending order according to the P-value; that is, the more significant the enrichment (the smaller the P-value), the higher the term (Fig. 6G). As a result, the 15 key MEM-related genes were primarily enriched in biological processes related to cellular aerobic respiration, such as "oxidative phosphorylation,” "NADH dehydrogenase complex assembly,” and "mitochondrial respiratory chain complex I assembly.” Their primary enriched cellular components were related to aerobic respiration, such as "mitochondrial respiratory chain complex I,” "NADH dehydrogenase complex,” and "respiratory chain complex I.” The KEGG analysis results showed that the primary enriched signaling pathways of the 15 MEM-related genes were connected to metabolism, such as “Oxidative phosphorylation,” “Ferroptosis,” and “Cholesterol metabolism” (Fig. 6H).

### 3.8 Molecular typing of TCGA-ESCC tumor samples based on key MEM genes

Based on the 15 key MEM-related genes and FPKM expression data from common transcriptome sequencing of TCGA-ESCC samples, ConsensusClusterPlus was used to classify the ESCC samples. The number of clusters was determined to be 2 by comprehensive consideration of the consistency matrix heatmap (Fig.S2A), a cumulative distribution curve (Fig. S2B) and a delta area curve (Fig. S2C). In both subtypes, the expression levels of *SPP1, APOE, BID, NDUFB2,* and *NEUFA13* were significantly different (P < 0.05, Fig. S2D). A t-SNE analysis was performed based on the 15 MEM-related genes, and the results showed that the two ESCC subtypes clustered into two distinct clusters (Fig. S2E).

In addition, ssGSEA revealed the differences in 16 immune cells (Fig.S2F) and the enrichment pathways of 13 immune cells in different molecular subtypes (Fig. S2G). Macrophages were more abundant in Cluster1 than in Cluster2 (P < 0.05). Among other immune cells and immune-related pathways, the enrichment activities were not significantly different between the two subtypes.

### 3.9 Gene model construction for prognosis

To evaluate correlations between the 15 key MEM-related genes and the prognosis of patients with ESCC, 56 of 92 TCGA-ESCC patients were randomly selected to form the training set and a univariate COX regression analysis was performed. Seven MEM-related genes (*BID, MAP1LC3A, KLF2, APOE, APPL1, NDUFA13,* and *TUBB*) were found to have a greater impact on ESCC prognosis (P < 0.3). Then, a fitting model was built using regression analysis (Fig. S3A), and cross-validation analysis determined that the best lambda value of the fitting model was 5 (Fig. S3B). From this, 5 MEM-related genes were identified. Finally, a Cox regression analysis was conducted using these 5 genes, and a risk model was constructed.

$$\mathrm{riskScore}=\left(0.626*\mathrm{MAP}1\mathrm{LC}3A\right)+(0.321*\mathrm{APOE})+(-1.645*\mathrm{APPL}1)+(1.809*\mathrm{NDUFA}13)$$

In this study, the median risk score was used to divide the patients into high- and low-risk groups. Kaplan-Meier curves showed that the overall survival rate of high-risk patients was lower than that of low-risk patients (P < 0.05; Fig. S3C). ROC analysis was also performed to verify prediction accuracy, demonstrating that the risk score was a good predictor of overall survival (OS) in patients with ESCC. At 1 and 2 years, the area under the curve (AUC) was 0.78 and 0.86, respectively (Fig. S3D). Finally, the remaining 36 TCGA-ESCC patients were included as the validation set. Similarly, patients in the high-risk groups had a poorer overall survival rate than those in the low-risk groups based on the Kaplan-Meier curves (p = 0.053, Fig. S3E). Based on the timeROC, the risk score predicted the OS of patients in the validation set similarly to the training set, and the AUC of 1-year and 2-year OS were 0.77 and 0.88, respectively (Fig. S3F). Moreover, among the 92 patients with TCGA-ESCC, the high-risk patients' OS was lower than that of low-risk patients (P < 0.05; Fig. S3G) and the AUC of the risk score for predicting OS was also relatively high (1-year: 0.63; 2-year: 0.82; Fig. S3H).

**Fig. 7:**
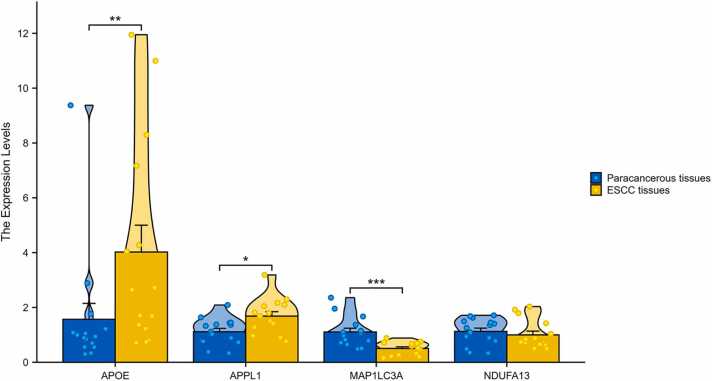
Expression levels of *APOE, APPL1, MAP1LC3A* and *NDUFA13* mRNA in ESCC tissues and paracancerous tissues from 15 patients. The results were shown with Mean ± SD. *p < 0.05; * **p < 0.001. SD, standard deviation.

The transcription levels of these four prognostic genes were further verified by RT-qPCR using a cDNA microarray from 15 patients with ESCC. There were greater expression levels of *APOE* and *APPL1* in ESCC tissues than in normal esophageal tissues, whereas the level of *MAP1LC3A* in ESCC tissues was lower (Fig. 7). The expression levels of *NDUFA13* did not differ significantly between the two tissues.

### 3.10 Correlation analysis of immune features

**Fig. 8:**
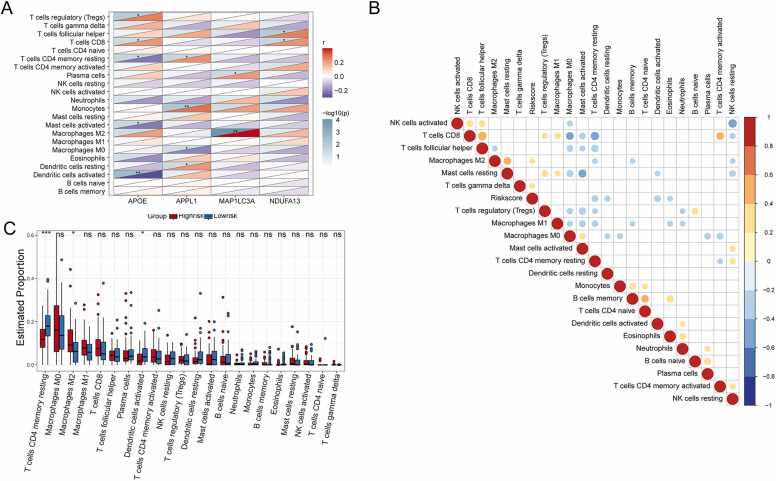
Immune signature analysis of prognostic MEM genes and risk scores based on TCGA-ESCC. **(A)** Heat map of correlation analysis between prognostic genes and infiltrating immune cells. **(B)** Heat map of correlation analysis between risk score and infiltrating immune cells. **(C)** Difference analysis of immune cell infiltration levels between high and low risk groups. (*: p < = 0.05, **: p < = 0.01, ***: p < = 0.001, ****: p < = 0.0001). MEM, mitochondrial energy metabolism.

To study the biological relationship between MEM genes and the immune microenvironment, immune cell infiltration in diseased samples was analyzed using the CIBERSORT script. Abundance values were obtained for 22 types of immune cells. Notably, the prognostic genes were closely related to some immune cells (Fig. 8A). For example, the abundance of plasma cells was positively correlated with *MAP1LC3A* (r = 0.210, P < 0.05), while that of T cells CD8 was positively correlated with *APOE* (r = 0.211, P < 0.05) and *NDUFA13* (r = 0.225, P < 0.05). Additionally, M2 macrophages were positively correlated with *MAP1LC3A* (r = 0.395, P < 0.05). The abundance of CD4 + memory resting T cells was negatively correlated with *APOE* (r = −0.226, P < 0.05) and positively correlated with *APPL1* (r = 0.225, P < 0.05). Activated DCs were negatively correlated with *APOE* (r = −0.308, P < 0.05). Furthermore, the correlation between risk score and immune cell abundance was examined (Fig. 8B). As a result, the risk score was negatively correlated with DC activation (r = −0.320, P < 0.05) and the resting memory of CD4 + T cells (r = −0.318, P < 0.05). The risk score was also positively correlated with M2 macrophages (r = 0.208, P < 0.05) and T cells gamma delta (r = 0.207, P < 0.05). A difference in immune cells was also observed between the high- and low-risk groups using a boxplot (Fig. 8C). The immune infiltration scores of CD4 memory resting T cells and activated DCs were significantly higher in low-risk samples than in high-risk samples, and the immune infiltration scores of M2 macrophages were significantly higher in high-risk samples than in low-risk samples (P < 0.05, Fig. 8C).

### 3.11 Impact of Risk Score on Genomic Changes in TCGA-ESCC Patients

**Fig. 9:**
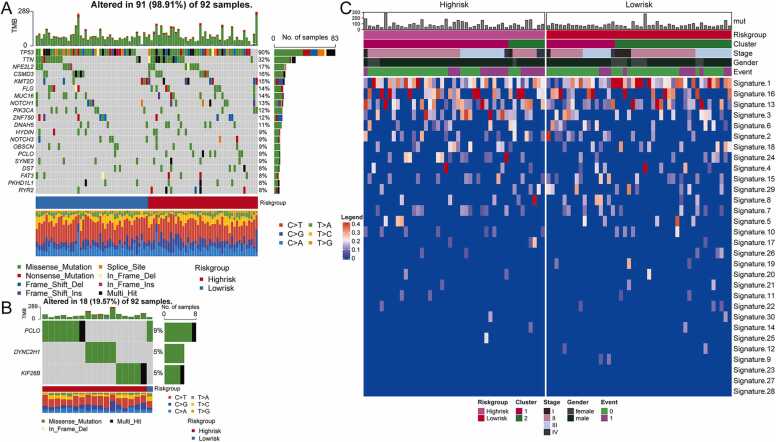
The influence of risk grouping based on TCGA-ESCC on the genetic variation of ESCC patients. **(A)** The waterfall chart showing the mutation status of the top 20 genes in the high- and low-risk groups. **(B)** Waterfall plot showing genes with significantly different mutations between high and low risk groups. **(C)** Heatmap showing mutation signatures between high and low risk groups.

To assess the effect of MEM-related risk scores on changes in genetic variation, including single nucleotide polymorphisms (SNPs) and copy number variations (CNVs), somatic mutation data was downloaded. First, the maftools package was used to display the mutations in the top20 genes in the TCGA-ESCC high- and low-risk groups, including TP53、TTN、NFE2L2、CSMD3、KMT2D、FLG、MUC16、NOTCH1、PIK3CA、ZNF750、DNAH5、HYDIN、NOTCH3、OBSCN、PCLO、SYNE2、DST、FAT3、PKHD1L1、RYR2 (Fig. 9A). Among the high-risk group and the low-risk group, the presence of *PCLO* mutations were significantly different (P < 0.05). In addition, genes with mutational differences between the high- and low-risk groups were identified (Fig. 9B). The results showed that in addition to *PCLO, DYNC2H1* and *KIF26B* also had significantly different mutation sample numbers between the high- and low-risk groups (P < 0.05).

Based on the median TMB, patients were divided into low- and high-TMB groups. In the two groups, the overall survival rates were not statistically different (Fig. S4A). Moreover, there was no statistical difference between high- and low-risk TMB groups (Fig. S4B), and there was no linear correlation with the risk score (Fig. S4C). Then, the MSI data of patients with ESCC was dowloaded, revealing that high- and low-risk MSI were not statistically different (Fig. S4D). Indeed, the risk score did not correlate linearly with the MSI (Fig. S4E).

Furthermore, the mutation signatures of TCGA-ESCC samples were visualized using the maftools package (Fig. 9C). The results showed that mutation signatures 1, 16, 13, 3, 6, 2, 18, and 24 occurred more frequently in the tumor samples than in the normal ones. Using GISTIC 2.0 and maftools, the CNV data of tumors was analyzed and visualiz, respectively (Fig. S5A–F). In the low-risk group, the gene copy number fragments that were significantly lost were 2q22.1, 8p23.2, and 9p21.3, while those with significant amplification were 7p11.2 and 11q13.3 (Fig. S5A). In the high-risk group, the gene copy number fragments were 2q22.1, 3p14.3, and 9p21.3, while those with significant amplification were 3q28 and 11q13.3 (Fig. S5B). Compared with the low-risk group, the fragments of gene copy number amplification (Fig. S5C–D) or deletion (Fig. S5E–F) in the high-risk group were significantly increased.

### 3.12 Evaluation of drug sensitivity in patients with ESCC based on MEM-related prognostic genes

To explore whether MEM-related prognostic genes could be used to evaluate the sensitivity of ESCC to different treatment drugs, the gene expression data of 60 cell lines and IC_50_ values of 24,360 drugs were downloaded from the CellMiner database. Excluding genes and drugs with incomplete information, we analyzed the correlation between the four MEM-related prognostic genes and the IC_50_ values of 62 drugs. There was a significant correlation between the IC_50_ values of 31 of these drugs and the prognostic genes (P < 0.05, Supplementary Table S16). *APOE* expression was positively correlated with the IC_50_ value of hypothemycin (r = 0.368, P < 0.05) and negatively correlated with that of 7 drugs, including batracylin (r = −0.391, P < 0.05) and perifosine (r = −0.345, P < 0.05). *APPL1* expression was positively correlated with the IC_50_ value of PX-316 (r = 0.306, P < 0.05). *MAP1LC3A* expression was positively correlated with the IC_50_ value of okadaic acid (r = 0.331, P < 0.05) and negatively correlated with that of 6 drugs, including 6-mercaptopurine (r = −0.412, P < 0.05) and 6-thioguanine (r = −0.378, P < 0.05). *NDUFA13* expression was positively correlated with the IC50 of 15 drugs, including hydroxyurea (r = 0.436, P < 0.05), melphalan (r = 429, P < 0.05), pipobroman (r = 0.413, P < 0.05), carboplatin (r = 0.408, P < 0.05), and chlorambucil (r = 0.400, P < 0.05). The correlations between the prognostic genes and certain drugs are displayed in Fig. S6.

**Fig. 10:**
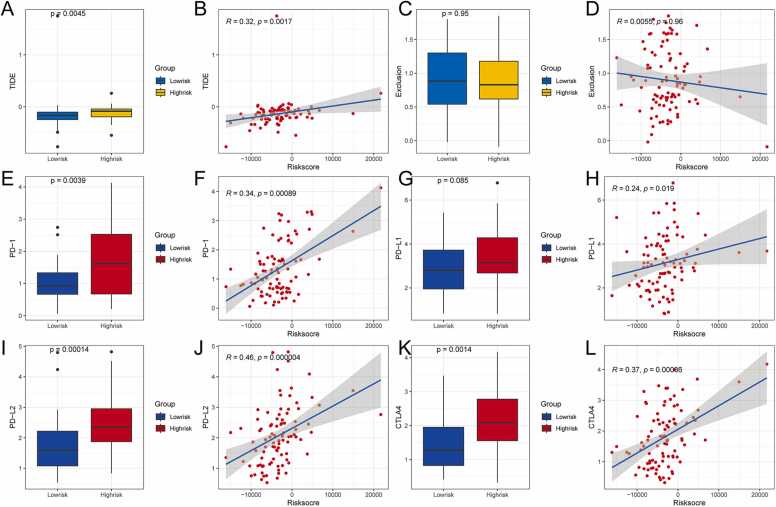
Difference analysis of TIDE scores and immune checkpoints in different risk groups. **(A)** Differences in TIDE across risk groups. **(B)** Correlation analysis between TIDE and risk score. **(C)** The difference of Exclusion among different risk groups. **(D)** Correlation analysis between Exclusion and risk score. **(E)** Differences in the expression of PD-1. **(F)** Correlation analysis between the expression of PD-1 and the risk score. **(G)** Differences in the expression of PD-L1 among different risk groups. **(H)** Correlation analysis between the expression of PD-L1 and the risk score. **(I)** Differences in the expression of PD-L2 among different risk groups. **(J)** Correlation analysis between the expression of PD-L2 and the risk score. **(K)** Differences in the expression of CTLA4 among different risk groups. **(L)** Correlation analysis between CTLA4 expression and risk score. TIDE, Tumor Immune Dysfunction and Exclusion.

Considering the importance of immunotherapy in treating tumors, the sensitivity of high- and low-risk patients to immunotherapy was evaluated using the TIDE algorithm. TIDE scores of the high-risk group were higher than those in the low-risk group (P < 0.05; Fig. 10A). Furthermore, TIDE score was also positively correlated with the risk score (R = 0.32, P < 0.05; Fig. 10B), suggesting that immunotherapy is less effective in high-risk patients. While an exclusive score usually reflects the strength of the immune escape response, the exclusive scores of the high- and low-risk groups did not differ significantly (P > 0.05; Fig. 10C), and there was no linear correlation with the risk score (P > 0.05; Fig. 10D).

To further assess the effectiveness of immunotherapy in patients with cancer, this study estimated the correlation between the common immune checkpoints PD1, PD-L1, PD-L2, CTLA4, and the risk scores (Fig. 10 E–L). The results showed that the expression of PD-1 in the high-risk group was higher than that in the low-risk group (P < 0.05, Fig. 10E), and was positively correlated with the risk score (r = 0.34, P < 0.05, Fig. 10F). There was no statistically significant difference in the expression of PD-L1 between the high-risk group and the low-risk group (P = 0.085, Fig. 10G), but it was positively correlated with the risk score (r = 0.24, P < 0.05, Fig. 10H). The expression of PD-L2 in the high-risk group was higher than that in the low-risk group (P < 0.05, Fig. 10I), and was positively correlated with the risk score (r = 0.46, P < 0.05, Fig. 10J). The expression of CTLA4 in the high-risk group was higher than that in the low-risk group (P < 0.05, Fig. 10K), and was positively correlated with the risk score (r = 0.37, P < 0.05, Fig. 10L). These results suggest that immunotherapy targeting these four immune checkpoints may be more sensitive in high-risk patients.

## 4 Discussion

Currently, the clinical outcomes of ESCC remain unsatisfactory, primarily owing to an insufficient understanding of its TME heterogeneity[2]. Tumor metabolism is primarily responsible for the formation of the TME, and metabolic reprogramming affects the efficacy of tumor immunotherapy [32]. Furthermore, MEM reprogramming is considered a hallmark of cancer because tumor cells need to maintain high metabolic rates, which are critical for cell growth and division[33]. Indeed, nebivolol targets MEM and angiogenesis to trigger a crisis in tumor cell metabolism and oxidative stress, thereby limiting tumor cell growth[34]. Therefore, the role of MEM in tumorigenesis may be exploited as a strategy for ESCC treatment. At present, the degree of cellular heterogeneity, dynamics of MEM, and their functional impact on the tumor ecosystem remain largely undefined in ESCC. Our study explored the metabolic state of immune cells in the ESCC microenvironment and constructed a promising prognostic signature based on MEM-related genes.

In the original study of dataset GSE145370[14], Zheng et al. proposed that T cells, NK cells, monocytes/macrophages, DCs, B cells, plasma cells, and mast cells were predominantly present in the ESCC tumor, suggesting an inflammatory and immunosuppressive TME (iTME). Here, our study was similar to the original study in terms of data processing. Indeed, the cell clusters we identified were highly consistent with their findings, and the marker genes of immune cells were highly expressed in the corresponding cell clusters, validating the existence of cellular heterogeneity in the iTME. Zheng et al. also proposed that mutual interference between macrophages and regulatory T cells (T regs) through ligand-receptor interactions may contribute to this immunosuppressive state and disease progression[14]. Consistent with this previous study, we found that macrophages and T cells, as MEM-high-scoring cell populations, had the strongest signaling communication, and their MEM states were significantly different. The construction of the iTME is closely related to MEM reprogramming of immune cells[35]. Therefore, our study not only verified the findings of Zheng et al., but also revealed the formation mechanism of the iTME.

We identified 121 MEM-related genes that were differentially expressed in seven cell populations. This suggests that there may be differences in MEM between immune and stromal cells, thereby affecting antitumor immune activity. We further found that macrophages, T cells, epithelial cells, and B cells were high-scoring cells expressing MEM-related genes, and their immune properties may be related to the top2 differential marker genes. Previous studies have shown that SPP1 + macrophages have immunosuppressive properties that prevent cytotoxic lymphocyte infiltration into the tumor core, serving as potential targets for tumor growth and metastasis [36], [37], [38]. Furthermore, macrophages with high expressions of *APOC1* and *SPP1* can limit the efficacy of anti-programmed cell death protein 1/ligand 1 (PD-1/ L1) therapy [39], [40]. The inhibition of *APOC1* expression activates immunity by reversing the macrophage M2 phenotype to the M1 phenotype via the ferroptotic pathway [40]. *GZMA* and *GNLY* are classic genes related to cytotoxicity in T cells [41]. There is a correlation between *IGKC+* B cell infiltration and patient prognosis [42]. GO- and KEGG-enrichment analyses showed that MEM-related genes were involved in immune regulation of the TME, as MEM affected the function and activity of immune cells. In addition, the expressions of 17 MEM-related marker genes were significantly higher in macrophages than in the other cells. This suggests that MEM-related genes are involved in the iTME in ESCC [14].

We found that the MEM state of T cells differed significantly from that of macrophages and epithelial cells. Overactive metabolism in tumor cells deprives T cells of the nutrients needed for function [43]. The energy metabolism of tumor-associated macrophages (TAMs) is dynamic. Their changes in response to the nutritional requirements of tumor cells not only have a strong impact on TAM survival, but also have a profound impact on tumor progression and tumor-targeted immune responses [44]. Additionally, cancerous areas have more epithelial cells than stromal areas. This is a strong indicator of ESCC [45]. Therefore, remodeling the MEM of T cells, macrophages, and epithelial cells in the TME to enhance antitumor immunity may be a novel therapeutic strategy.

Intercellular receptor-ligand pairs can elucidate cellular state-specific signaling relationships. The *SPP1-CD44* interaction is critical in the TME, as it suppresses T cell activation and promotes metastasis [36]. The interaction of *MIF* with *CD74* activates the MAPK pathway and inhibits the p53 pathway, leading to tumor cell growth[46]. Antigen-presenting cells (APCs) present tumor-associated antigens to *CD8 +* T cells through MHC class I molecules such as *HLA-B.* They also present antigens to *CD4 +* T cells through MHC class II molecules, including *HLA-DPA1, HLA-DPB1, HLA-DRA,* and *HLA-DRB1*, thereby eliciting antitumor immune responses [47]. A recent study identified unique long-lasting antigen-specific synaptic interactions between macrophages and *CD8 +* T cells in the TME [48]. Although these interactions fail to activate T cells, they leave them in an exhausted state. This process is accelerated under hypoxic conditions to protect the tumors. These results indicate that immunosuppressive macrophages frequently crosstalk with T cells, which may inhibit their activation and infiltration of T cells. This may be related to a significant difference in the MEM between the two cell types, which may be one of the mechanisms underlying iTME formation.

ESCC occurs and develops in close association with the mitochondrial metabolic reprogramming of tumor cells [11]. We found that the marker MEM-related genes were also differentially expressed in tumors and adjacent tissues, potentially indicating new therapeutic targets. Restricting the metabolic regulation of tumor cells and placing them into a specific metabolic state through drug therapy combined with changes in nutrient supply may impede ESCC progression and improve therapeutic efficacy [49]. We constructed a 4-gene prognostic model. The risk score had a good predictive ability for OS. Additionally, *MAP1LC3A, APOE,* and *NDUFA13* expressions were positively associated with high risk, whereas *APPL1* was negatively associated. Bioinformatics analysis revealed that *APOE* was highly expressed in ESCC, whereas *MAP1LC3A, APPL1,* and *NDUFA13* were lowly expressed. Moreover, RT-qPCR experiments based on the cDNA microarrays of 15 patients with ESCC verified the expression trends of *APOE* and *MAP1LC3A* at the transcriptional level, whereas those of *APPL1* and *NDUFA13* were not. Therefore, *APOE* and *MAP1LC3A* are potential target MEM-related genes for the development of anti-ESCC drugs.

*APOC1 +APOE+* TAMs promote distant metastasis of ESCC by aiding tumor colonization in the lymph nodes through matrix reorganization, collagen deposition, and MEM [50]. A previous study has shown that prostate cancer cells promote the senescence of *TREM2 +* immunosuppressive neutrophils by secreting *APOE*, which exerts immunosuppressive and tumor-promoting effects. The *APOE/TREM2* axis is associated with poor prognosis [51]. Therefore, our findings regarding APOE are consistent with those of the aforementioned studies.

In many human cancer cell lines, MAP1LC3A, a key molecule for mitophagy, is transcriptionally inactivated. This inactivation is caused by aberrant DNA methylation. Restoration of *MAP1LC3A* expression in ESCC cell lines led to inhibition of tumor growth in vivo [52]. *MAP1LC3A* expression is inversely correlated with the histological grade and aggressiveness of lung cancers. However, *MAP1LC3A* expression is positively correlated with high proliferative characteristics of tumor cells. High metabolic rates and ROS levels are positively associated with *MAP1LC3A* [53]. This is consistent with our findings that low *MAP1LC3A* expression in ESCC may be related to the aggressiveness of malignant cells, whereas high *MAP1LC3A* expression increases the risk of poor prognosis and may be related to the proliferation of tumor cells.

Low expression of *APPL1* may adapt to tumor malignant progression by reducing oxygen-consuming metabolism and regulate tumor immune infiltration and chemotherapy resistance [54]. *NDUFA13* is a subunit of complex I in the mitochondrial respiratory chain. It may be stimulated by IFN-b/RA to translocate from the mitochondria to the nucleus to initiate apoptosis [55].

There was a negative correlation between the risk score and *CD4 +* T cells and DCs and a positive correlation between the risk score and M2 macrophages. *CD4 +* T-cell interactions with DCs and enhanced antigen cross-presentation can enhance antitumor cytotoxic *CD8 +* T-cell responses [56]. M2 macrophages are immunosuppressive and promote tissue repair and tumorigenesis [39], [40]. Therefore, iTME is one of the reasons for the poor prognosis in high-risk patients. These results suggest that these four MEM-related genes have the potential to be immunotherapeutic targets for remodeling the TME, reducing the risk level, and improving prognosis.

Risk scores also contribute to changes in the levels of genetic variations, including SNPs and CNVs, in patients with ESCC. The mutation levels of *PCLO, DYNC2H1,* and *KIF26B* were significantly higher in the high-risk group. High-risk patients exhibited significantly higher CNV levels than those of low-risk patients. These results can help us better understand the molecular mechanisms by which MEM genes affect prognosis and may aid in the development of targeted pharmacotherapies.

We found that the expression of prognostic genes significantly correlated with the IC_50_ values of 31 drugs; therefore, they could be used to evaluate the sensitivity of ESCC to various drug treatments. Furthermore, the TIDE scores indicate that high-risk patients respond poorly to immunotherapy. However, PD-1, PD-L1, PD-L2, and CTLA4 levels were positively correlated with the risk score. This suggests that immunotherapy targeting the above four immune checkpoints may have a higher efficacy in high-risk patients. In summary, the prognostic signature showed promising results in predicting the therapeutic effects within the study cohort and may have potential clinical utility, helping personalize treatment. However, further validation and testing in larger cohorts or clinical settings are required to determine their clinical applicability.

To our knowledge, our study is the first to establish a prognostic signature for patients with ESCC based on MEM-related genes. However, we did not collect new ESCCs for scRNA sequencing to verify the expression of MEM marker genes. Secondly, the lack of age and sex details in the GSE111011 and GSE145370 datasets prevented us from performing a subgroup analysis to investigate whether the different subgroups exhibited differential expression of MEM-related marker genes. Thirdly, the two markers we found may not demonstrate complete specificity for B-cells. Future studies could potentially explore additional markers or combinations of markers to further enhance the specificity and accuracy of B-cell identification. Fourthly, we did not investigate the specific mechanisms through which cellular crosstalk leads to distinct MEM states. Moreover, the efficacy of the proposed precision medicine must be verified in a laboratory.

## 5 Conclusion

MEM reprogramming is an important hallmark of ESCC development. Based on scRNA-seq data, this study revealed the expression of 121 marker MEM-related genes in different cell populations of the ESCC microenvironment and identified four high-scoring cell populations. The MEM state of T cells is significantly different from that of macrophages and epithelial cells, and signaling communication between T cells and macrophages is the strongest. These findings suggest that immunosuppression is related to metabolic reprogramming. Marker genes of high-scoring cells and top10 receptor-ligand pairs may become new targets for rebuilding immune cell metabolism. The 4-MEM gene risk signature showed good predictive power for OS and drug sensitivity. RT-qPCR based on cDNA microarrays from 15 patients with ESCC validated the expression trends of *APOE* and *MAP1LC3A* at the transcriptional level. Therefore, this model provides guidance for clinical practice, facilitates the screening of early treatment patient populations, and helps realize personalized treatments.

## Ethics Approval and Consent to Participate

The cDNA microarray was approved by the ethics committee of Shanghai Outdo Biotech Company (Shanghai, China) (approval number: YB M-05–02).

## Permission Note

Not applicable.

## Consent for publication

Not applicable.

## Funding

This work was supported by the 2022 Science and Technology Plan Projects of Ordos Municipal Bureau of Science and Technology, Ordos, Inner Mongolia Autonomous of China (No. 2022YY014), the 10.13039/501100001809Natural Science Foundation of China (No.81874220) and the 10.13039/501100003453Natural Science Foundation of Guangdong Province (No.2020A1515010030).

## CRediT authorship contribution statement

ZL and L-QF contributed to concept and design of this study. Z-ZW, J-GW and ZJ wrote the manuscript. Collecting and analyzing data was done by Z-ZW, ZJ, D-SQ, CF, W-GW. Data analysis and interpretation were performed by Z-ZW, J-GW, L-QF and ZL. This article was written by all authors, and version submitted by all authors has been approved.

## Declaration of Competing Interest

none.

## Data Availability

Articles/supplementary materials containing the original contributions are included in this study; please contact the corresponding author with any further questions.

## Appendix A Supplementary material

**Table S1.** Summary of the information on the GEO datasets. GEO, Gene Expression Omnibus.

.

**Table S10.** Differential genes among high-scoring cell populations.

.

**Table S11.** Gene Ontology (GO) enrichment analysis of differential genes among high-scoring cell populations.

.

**Table S12.** Receptor-ligand pairs among high-scoring cell populations.

.

**Table S13.** Differences between tumor tissues and normal tissues of 121 mitochondrial energy metabolism-related genes in the GSE111011 dataset.

.

**Table S14.** Differences between tumor tissues and normal tissues of 121 mitochondrial energy metabolism-related genes in the GSE164158 dataset.

.

**Table S15.** Gene Ontology (GO) enrichment analysis of 15 key genes of mitochondrial energy metabolism.

.

**Table S16.** Correlation analysis between IC50 values of 31 drugs and prognostic genes

.

**Table S2.** Baseline table of clinical data for the SCC dataset in the TCGA-ESCA dataset.

.

**Figure S1.** Flowchart of the study design. TCGA: The Cancer Genome Atlas; ESCC: esophageal squamous cell carcinoma; GO: Gene Ontology; KEGG: Kyoto Encyclopedia of Genes and Genomes; LASSO: Least absolute shrinkage and selection operator; MEMRGs: mitochondrial energy metabolism related genes.

.

**Figure S2.** Identification and analysis of tumor subtypes based on TCGA-ESCC. **(A)** Consistency clustering matrix heat map. **(B)** CDF plot. **(C)** Delta Area Plot. **(D)** Box plot of the expression levels of 15 key MEM genes in different tumor subtypes. **(E)** t-SNE distribution based on key MEM genes. **(F)** Degree of Immune Cell Infiltration. **(G)** Activity of immune-related pathways in different tumor subtypes. (*: p < = 0.05, **: p < = 0.01, ***: p < = 0.001, ****: p < = 0.0001). CDF, cumulative distribution function; MEM, mitochondrial energy metabolism; t-SNE, t-distributed stochastic neighborhood embedding.

.

**Figure S3.** Correlation of key MEM genes with ESCC prognosis based on TCGA-ESCC. **(A)** LASSO shows the change of lambda value of 7 MEM genes with significant prognosis. **(B)** Cross-validation analysis to determine the best value of lambda for the fitted model. **(C)** Survival curves of TCGA-ESCC training set. **(D)** ROC curves for OS of the TCGA-ESCC training set. **(E)** Survival curves of the TCGA-ESCC validation set. **(F)** ROC curves of OS in TCGA-ESCC validation set. **(G)** Survival curves of TCGA-ESCC. **(H)** ROC curves of TCGA-ESCC overall survival. A p value < 0.05 was considered significant difference. MEM, mitochondrial energy metabolism.

.

**Figure S4.** Comparison of tumor mutational burden and microsatellite instability between high and low risk groups. **(A)** Survival analysis between high TMB patients and low TMB patients. **(B)** Comparison of TMB between high and low risk groups. **(C)** Correlation analysis of TMB and risk score. **(D)** Comparison of MSI between high and low risk groups. **(E)** Correlation analysis between MSI and risk score. TMB, tumor mutation burden; MSI, microsatellite instability score.

.

**Figure S5.** Analysis of copy number variation in different risk groups based on TCGA-ESCC. **(A-B)** Gene fragments with significant copy number variation in the low-risk group and high-risk group. **(C-D)** Gene segments with increased copy number in the low-risk group and high-risk group. **(E-F)** Gene segments with copy number deletions in the low-risk group and high-risk group.

.

**Figure S6.** Correlation analysis between prognostic genes and drug IC50. **(A)***APOE* was positively correlated with IC50 of HYPOTHEMYCIN. **(B)***APOE* was negatively correlated with IC50 of Batracylin. **(C)***APPL1* was positively correlated with IC50 of PX-316. **(D)***MAP1LC3A* was positively correlated with IC50 of Okadaic acid. **(E)***MAP1LC3A* was negatively correlated with IC50 of 6-MERCAPTOPURINE. **(F)***NDUFA13* was positively correlated with IC50 of Hydroxyurea. **(G)***NDUFA13* was positively correlated with IC50 of Melphalan. **(H)***NDUFA13* was positively correlated with IC50 of Pipobroman.

.

**Table S3.** 599 mitochondrial energy metabolism-related gene sets. SCC, squamous cell carcinoma.

.

**Table S4.** Detailed pathological data of the cDNA microarray (MecDNA-HEsoS030PG01)

.

**Table S5.** Information of primers.

.

**Table S6.** 16 immune cell-related gene sets.

.

**Table S7.** 13 immune pathway-related gene sets.

.

**Table S8.** Differential genes among different cell types.

.

**Table S9.** Correlation coefficients of 20 mitochondrial energy metabolism-related genes
