# Supplementary figures and images for: Mitochondrial energy metabolism correlates with an immunosuppressive tumor microenvironment and poor prognosis in esophageal squamous cell carcinoma
Source: Comput Struct Biotechnol J. 2023 Aug 24;21:4118–33. doi: 10.1016/j.csbj.2023.08.022 (PMC10474161; doi:10.1016/j.csbj.2023.08.022)

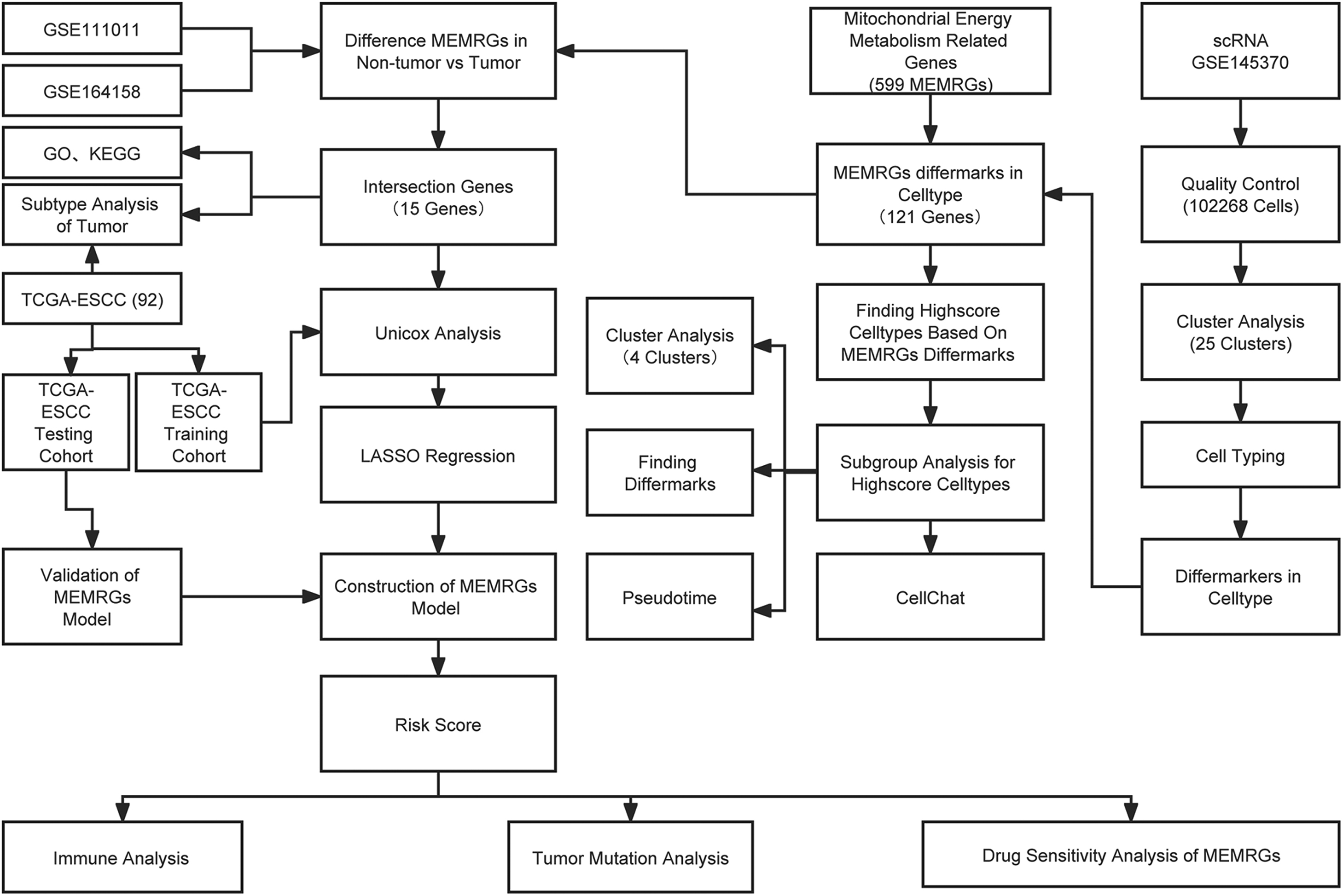

Supplement: Supplementary file 10 — Figure S1. Flowchart of the study design. TCGA: The Cancer Genome Atlas; ESCC: esophageal squamous cell carcinoma; GO: Gene Ontology; KEGG: Kyoto Encyclopedia of Genes and Genomes; LASSO: Least absolute shrinkage and selection operator; MEMRGs: mitochondrial energy metabolism related genes. [file mmc17.jpg]

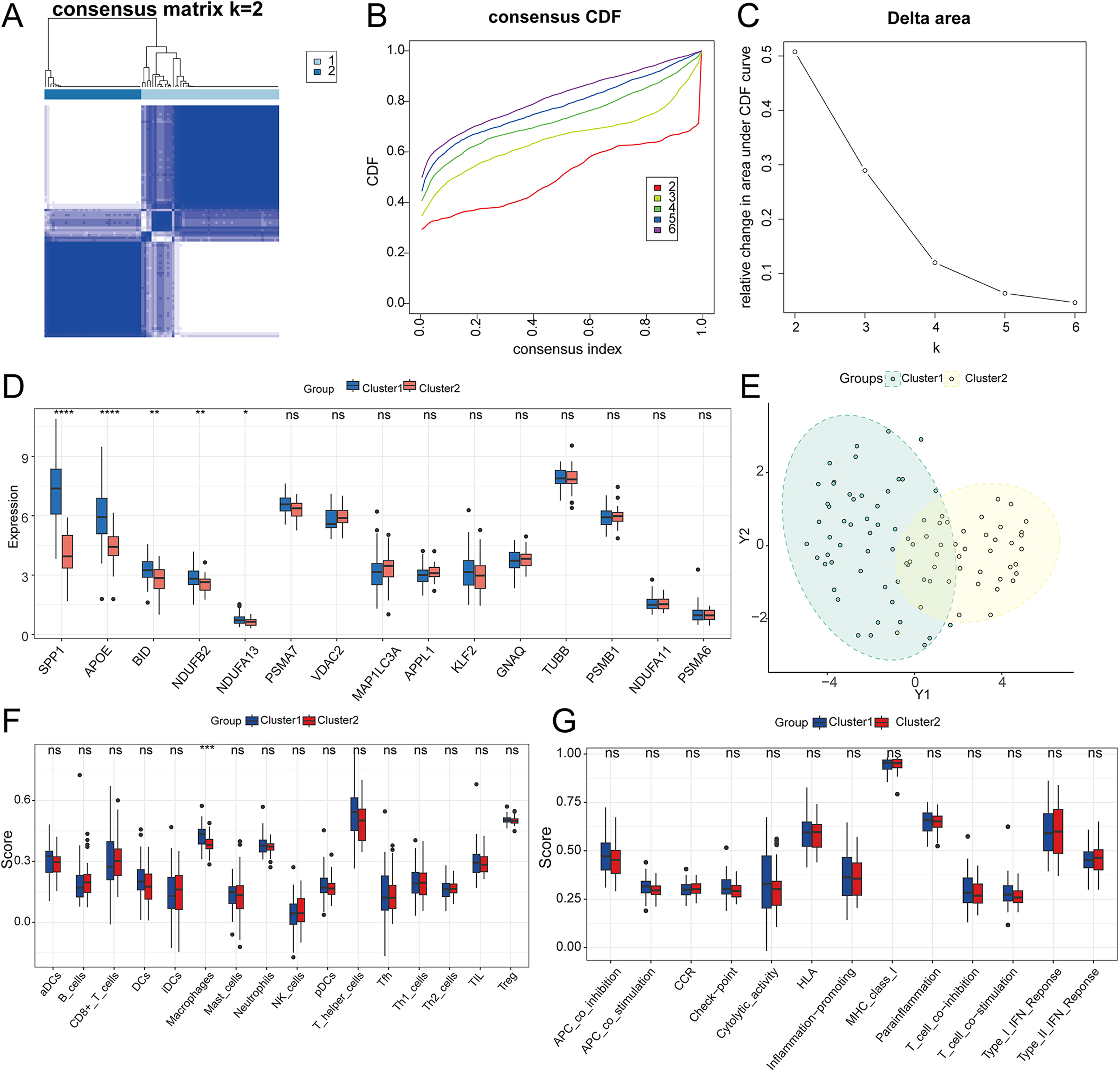

Supplement: Supplementary file 11 — Figure S2. Identification and analysis of tumor subtypes based on TCGA-ESCC. (A) Consistency clustering matrix heat map. (B) CDF plot. (C) Delta Area Plot. (D) Box plot of the expression levels of 15 key MEM genes in different tumor subtypes. (E) t-SNE distribution based on key MEM genes. (F) Degree of Immune Cell Infiltration. (G) Activity of immune-related pathways in different tumor subtypes. (*: p < = 0.05, **: p < = 0.01, ***: p < = 0.001, ****: p < = 0.0001). CDF, cumulative distribution function; MEM, mitochondrial energy metabolism; t-SNE, t-distributed stochastic neighborhood embedding. [file mmc18.jpg]

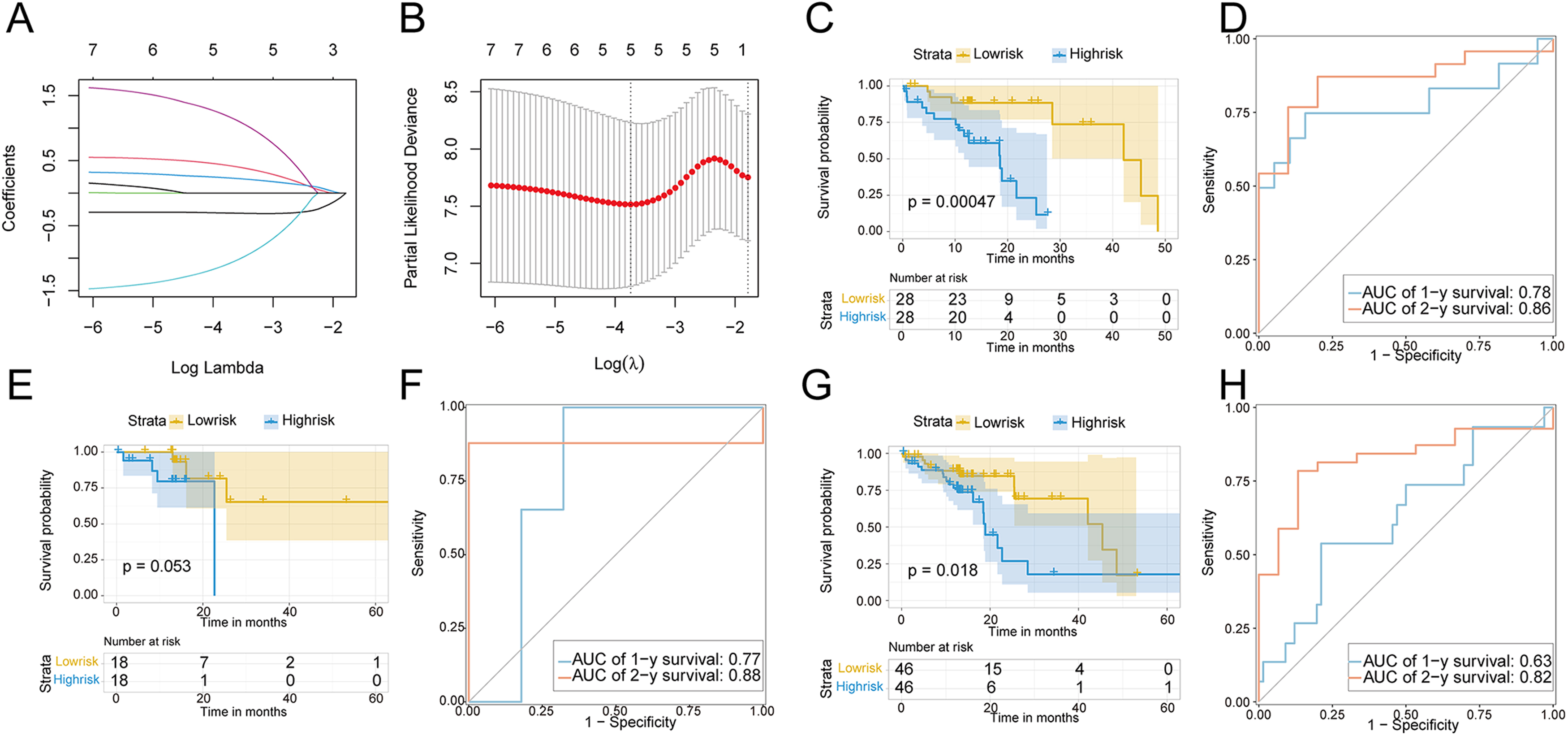

Supplement: Supplementary file 12 — Figure S3. Correlation of key MEM genes with ESCC prognosis based on TCGA-ESCC. (A) LASSO shows the change of lambda value of 7 MEM genes with significant prognosis. (B) Cross-validation analysis to determine the best value of lambda for the fitted model. (C) Survival curves of TCGA-ESCC training set. (D) ROC curves for OS of the TCGA-ESCC training set. (E) Survival curves of the TCGA-ESCC validation set. (F) ROC curves of OS in TCGA-ESCC validation set. (G) Survival curves of TCGA-ESCC. (H) ROC curves of TCGA-ESCC overall survival. A p value < 0.05 was considered significant difference. MEM, mitochondrial energy metabolism. [file mmc19.jpg]

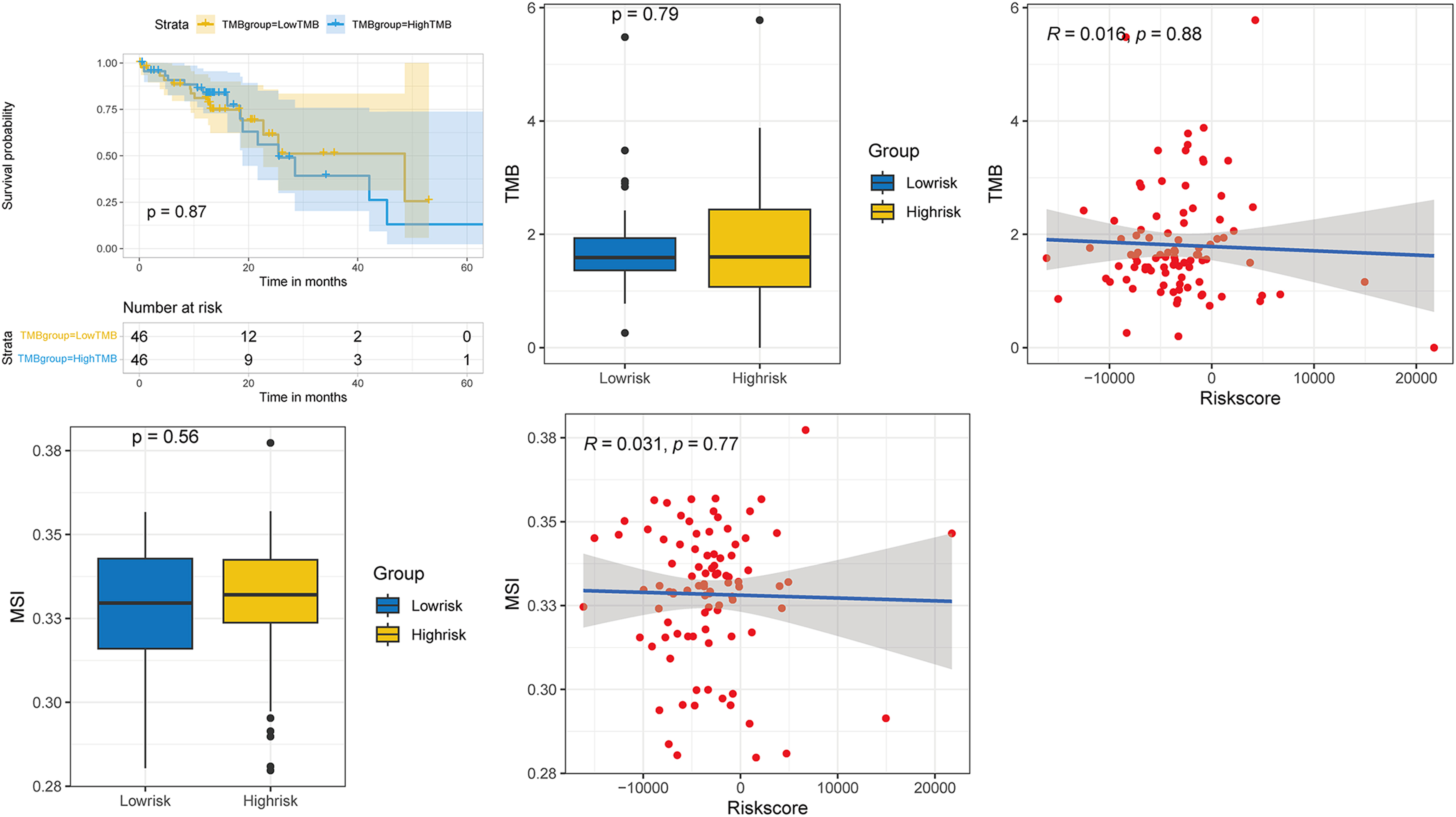

Supplement: Supplementary file 13 — Figure S4. Comparison of tumor mutational burden and microsatellite instability between high and low risk groups. (A) Survival analysis between high TMB patients and low TMB patients. (B) Comparison of TMB between high and low risk groups. (C) Correlation analysis of TMB and risk score. (D) Comparison of MSI between high and low risk groups. (E) Correlation analysis between MSI and risk score. TMB, tumor mutation burden; MSI, microsatellite instability score. [file mmc20.jpg]

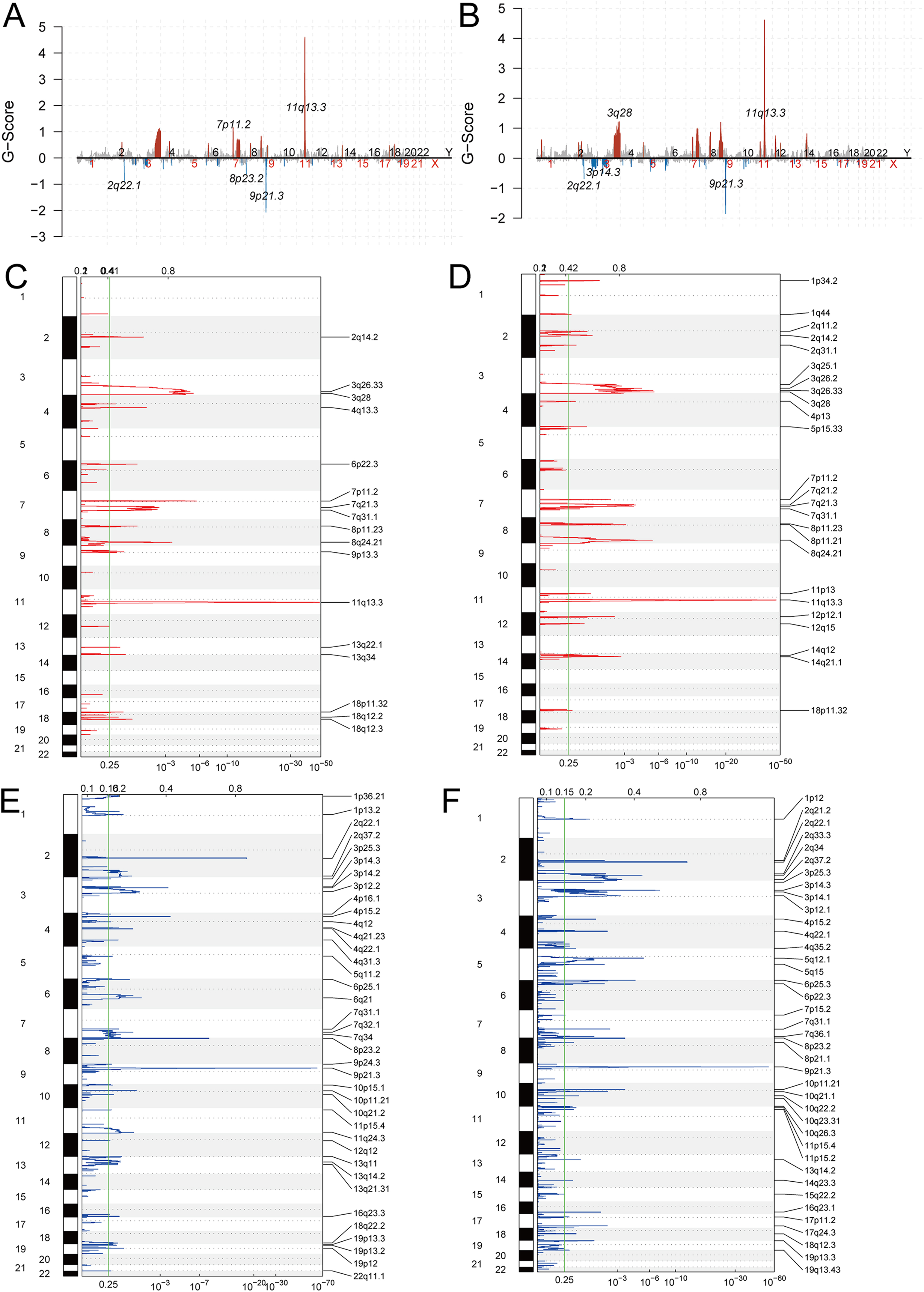

Supplement: Supplementary file 14 — Figure S5. Analysis of copy number variation in different risk groups based on TCGA-ESCC. (A-B) Gene fragments with significant copy number variation in the low-risk group and high-risk group. (C-D) Gene segments with increased copy number in the low-risk group and high-risk group. (E-F) Gene segments with copy number deletions in the low-risk group and high-risk group. [file mmc21.jpg]

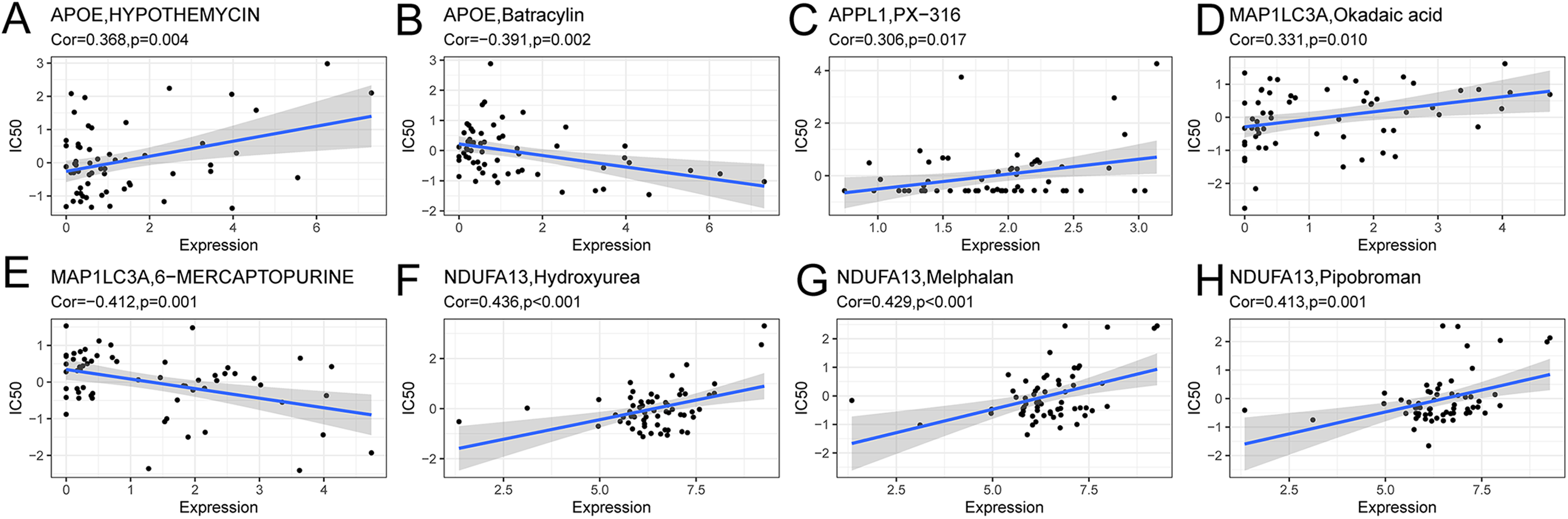

Supplement: Supplementary file 15 — Figure S6. Correlation analysis between prognostic genes and drug IC50. (A)APOE was positively correlated with IC50 of HYPOTHEMYCIN. (B)APOE was negatively correlated with IC50 of Batracylin. (C)APPL1 was positively correlated with IC50 of PX-316. (D)MAP1LC3A was positively correlated with IC50 of Okadaic acid. (E)MAP1LC3A was negatively correlated with IC50 of 6-MERCAPTOPURINE. (F)NDUFA13 was positively correlated with IC50 of Hydroxyurea. (G)NDUFA13 was positively correlated with IC50 of Melphalan. (H)NDUFA13 was positively correlated with IC50 of Pipobroman. [file mmc22.jpg]
